# Impact and Diagnostic Gaps of Comprehensive Genomic Profiling in Real-World Clinical Practice

**DOI:** 10.3390/cancers12051156

**Published:** 2020-05-04

**Authors:** Aditi P. Singh, Elaine Shum, Lakshmi Rajdev, Haiying Cheng, Sanjay Goel, Roman Perez-Soler, Balazs Halmos

**Affiliations:** 1 Division of Hematology and Oncology, University of Pennsylvania/Abramson Cancer Center, Philadelphia, PA 19104, USA; aditi.singh@pennmedicine.upenn.edu; 2 Division of Medical Oncology and Hematology, NYU Langone Perlmutter Cancer Center, New York, NY 10016, USA; elaine.shum@nyumc.org; 3 Department of Oncology, Montefiore Medical Center/Albert Einstein College of Medicine, Bronx, NY 10467, USA; lrajdev@montefiore.org (L.R.); hcheng@montefiore.org (H.C.); sgoel@montefiore.org (S.G.); rperezso@montefiore.org (R.P.-S.)

**Keywords:** comprehensive genomic profiling, molecular genotyping

## Abstract

Purpose: next-generation sequencing based comprehensive genomic profiling (CGP) is becoming common practice. Although numerous studies have shown its feasibility to identify actionable genomic alterations in most patients, its clinical impact as part of routine management across all cancers in the community remains unknown. Methods: we conducted a retrospective study of all patients that underwent CGP as part of routine cancer management from January 2013 to June 2017 at an academic community-based NCI-designated cancer center. CGP was done in addition to established first tier reflex molecular testing as per national guidelines (e.g., *EGFR*/*ALK* for non-small cell lung cancer (NSCLC) and extended-*RAS* for colorectal cancer). Results: 349 tests were sent for CGP from 333 patients and 95% had at least one actionable genomic alteration reported. According to the reported results, 23.2% had a Food and Drug Administration (FDA) approved therapy available, 61.3% had an off-label therapy available and 77.9% were potentially eligible for a clinical trial. Treatment recommendations were also reviewed within the OncoKB database and 47% of them were not clinically validated therapies. The CGP results led to treatment change in only 35 patients (10%), most commonly in NSCLC. Nineteen of these patients (54% of those treated and 5% of total) had documented clinical benefit with targeted therapy. Conclusion: we demonstrate that routine use of CGP in the community across all cancer types detects potentially actionable genomic alterations in a majority of patients, however has modest clinical impact enriched in the NSCLC subset.

## 1. Introduction

Targeted therapy against driver genomic alterations has improved outcomes for patients with many different cancers, including lung cancer, melanoma, breast cancer, and others [1]. Next-generation sequencing (NGS) based tumor comprehensive genomic profiling (CGP) that detects all classes of genomic aberrations (base pair substitutions, copy number variations, insertions/deletions, and rearrangements) is increasingly being utilized to match patients to relevant targeted therapies against several oncogenic drivers [2,3]. The National Comprehensive Cancer Network (NCCN) guidelines recommend “broad molecular profiling”, including *BRAF*, *ERBB2 (HER2)*, *MET*, *RET*, NTRK, and *ROS1*, in addition to *EGFR* and *ALK* for metastatic non-small cell lung cancer (NSCLC) [4,5,6,7]. Several in-house as well as commercial testing panels are now available with rapid turnaround times for results [1,8,9,10]. Several NGS-based platforms are being utilized in the care of cancer patients, since the Food and Drug Administration (FDA) approval of two NGS-based assays in November 2017 for patients with advanced stage cancer and the national coverage determination of the tests by Centers for Medicare and Medicaid Services (CMS) [11,12]. 

Despite guidelines, the uptake of CGP in the community has not been uniform, even in NSCLC patients and the general impact of CGP as to patient outcomes and cost effectiveness remains unclear [13,14]. A large retrospective study of advanced NSCLC patients treated in the community setting identified gaps in national guideline based genomic testing for *EGFR* and *ALK* [4]. Of 814 patients, 479 (59%) met guideline recommendations for *EGFR* and *ALK* testing and only 63 (8%) underwent testing for all eight NCCN recommended genomic alterations. The barriers cited for under-genotyping included sample handling issues, long turnaround times, confusion about test reimbursement, access to targeted therapies, and insufficient tissue. 

Several studies, mostly from large academic centers, have reported successful implementation of CGP and have shown that most patients will have at least one potentially actionable genomic alteration on CGP. In a retrospective study of 125 patients who underwent CGP, clinically relevant genetic alterations were found in 111 (92%) patients [15]. Only 15 (12%) patients received molecularly targeted therapy, with three who derived clinical benefit. The most common reasons for not receiving targeted therapy were ongoing standard of care treatment, poor performance status, stable disease, and lack of access to clinical trials. This trial was smaller than our study, included both adult and pediatric cases, mostly included brain tumors and assessed patients prior to 2016. A prospective trial of 100 patients with rare and/or refractory cancers assessed the clinical actionability of CGP, as determined by recommendations by a molecular tumor board [10]. Ninety-two patients underwent successful genetic sequencing and 96% (*n* = 88) had at least one genetic alteration. CGP led to change in management in 31% of patients, including targeted therapy, change in diagnosis, and germline testing. However, some of the cases included in this subset were those treated with cytotoxic chemotherapy, due to lack of driver mutations, e.g., a pancreatic tumor with *STK11* mutation treated with pemetrexed. Barriers to change in management were deteriorating patient clinical status and a lack of access to relevant clinical trials. Another prospective study assessed the feasibility of implementing CGP for all cancer patients at the institution and reported the results for the first 3727 patients who were successfully sequenced with their in-house gene panel [1]. Seventy-three percent of cases had at least one clinically actionable genetic alteration and only 19% of these were standard of care therapeutic recommendations at the time. However, this study did not look at actual change in management. A prospective, single arm study enrolled 500 patients with refractory cancers from a phase 1 oncology clinic, of which 339 patients underwent CGP [16]. Of these patients, 317 (93.5%) had at least one potentially actionable molecular alteration. The matching scores were calculated based on the number of drug matches and genomic alterations per patient. 122 of total 500 (24.4%) patients received matched therapy and 66 of 500 (13.2%) received unmatched therapy. High matching scores were associated with a greater frequency of stable disease, partial or complete remission versus low scores (22% vs. 9% respectively, *p* = 0.024), as well as longer survival (Hazard Ratio = 0.65; *p* = 0.05). A subsequent analysis of the same patient cohort found that patients on matched therapy had longer time on treatment (1.5 months), longer survival by 2.4 months, and higher drug treatment costs (by $38K) (*p* < 0.01) [2]. Sixty-six percent of increased drug costs were attributable to longer treatment time, as opposed to higher monthly drug costs. Patients who received matched therapies as an earlier line of treatment (1–3) derived more numerical improvement in the aforementioned areas when compared to those who received them as later line of therapy (4 and above). Although this study provided interesting results, its definition for “matched” therapies was quite liberal—including any drug that had a half maximal inhibitory concentration (IC50) in the low nanomolar range or if the target was the primary one recognized by an antibody. A retrospective review of 439 patients who underwent CGP showed that 393 (90%) patients had at least one potentially actionable molecular alteration [17]. The alteration was targetable by at least an experimental drug in a clinical trial in all cases. The drug was only FDA approved for their tumor (on-label) in 89 patients (20%), requiring off-label use for most recommended drugs. Again, this study did not assess actual change in management.

Recently, another study estimated that 8% of cancer patients in the United States were eligible for genome-targeted therapy and only 4.9% were estimated to actually derive benefit from this therapy (i.e., responders) [18]. The MOSCATO-1 trial prospectively enrolled 1035 patients with advanced cancers, of which 843 (89%) underwent successful molecular profiling [19]. 411 patients (48.7%) had actionable targets and 199 received matched therapy. The outcomes improved (assessed by PFS2/PFS1 score) in 63 patients (7% of total 843 screened) and objective responses were observed in 22 of all 1035 patients (2.1%). 

Our study aims to determine the real-world impact of routine incorporation of CGP in the community across all cancer types, regardless of stage or prior lines of therapy, while these studies have shed light on the feasibility as well as actionability in advanced cancers after standard treatments in the setting of clinical trials.

## 2. Methods

We conducted a retrospective, observational study of all patients that underwent comprehensive genomic profiling from January 1, 2013 to June 30, 2017 at an academic community-based National Cancer Institute (NCI)-designated cancer center. The institution has a multidisciplinary molecular tumor board that was established in September 2015, where some of these cases were referred and reviewed. CGP was performed either on tumor or plasma samples. Our institution, like many others, has a two-tier algorithm for molecular testing. The first-tier included tests that are reflexively sent by the Pathology department based on histology according to established institutional guidelines following national recommendations. For example, this included during the study era extended *RAS* testing for colorectal cancer, *HER2* for breast and esophagogastric cancer, and *EGFR/ALK* for lung adenocarcinoma. CGP constitutes the second tier and these tests are sent at the discretion of the treating oncologist. The assays used for NGS-based genomic profiling included the commercially available Foundation Medicine (Cambridge, MA, USA), Guardant Health (Redwood City, CA, USA), or Genoptix (Carlsbad, CA, USA) multi-gene panels. Testing facilities reported results defining potentially clinically actionable genetic alterations and listed treatment options available in three categories: FDA-approved on-label therapies, FDA-approved off-label therapies, and available clinical trials. Management change included patients in whom targeted therapy was initiated, continued, or withheld based on the results of CGP. Those who had clarification or confirmation of primary tumor or were enrolled on clinical trials based on CGP were also included. Patients already on targeted therapy based on previously known genetic alterations obtained by sequential or first-tier reflex testing were not included. Two independent physicians (A.S. and E.S.) reviewed all patient charts to obtain demographic as well as clinical data. 

Next, we utilized the publicly available precision oncology database, OncoKB [20] to assess the supportive evidence behind treatment recommendations for “actionable mutations” labeled by testing platforms. According to the database, each potentially clinically actionable genetic alteration was assigned a level of evidence if applicable. Level 1 denotes FDA-approved targeted therapy available, level 2 denotes the standard of care, but no FDA approved indication, level 3 and 4 are assigned to alterations where therapies are not standard of care, but have compelling clinical or biologic evidence for hypothetical benefit, respectively. Level R1 indicates a standard of care biomarker predictive of resistance to an FDA-approved therapy. In cases where no level of evidence is assigned, alterations are classified as oncogenic, likely oncogenic, oncogenic function unknown, or no information available. All patient information was de-identified. Descriptive statistics and chi-squared analyses were performed using Microsoft Excel. The Institutional Review Board (IRB) of Albert Einstein College of Medicine approved the study (IRB number: 2013-2570). 

## 3. Results

Three hundred and forty-nine tests were sent from 333 adult patients. The median age of patients was 63 years (range 19–98 years). One hundred and ninety-two (56%) of the patients were female. 112 (32%) patients were black and 71 (20.3%) identified as Hispanic. One hundred and twenty-four patients (63.4%) had Medicare or Medicaid and 26% had private insurance. The most common diagnosis for which CGP was sent was NSCLC (*n* = 107, 31.5%,), followed-by colorectal cancer (*n* = 58, 16%), ovarian cancer (*n* = 15, 4.3%), and carcinoma of unknown primary (*n* = 14, 4%) (a list of all primary oncologic diagnoses is listed in Table 1 in the appendix). 79.9% (*n* = 279) of tests were sent on tumor tissue samples and 20.1% (*n* = 70) were sent on plasma samples. The median number of therapies received for metastatic disease at the time of testing was 1 (range 0–5) and the median turnaround time for results was 12 days (range, 6–304 days). In seven cases, the results were not reported due to failed sequencing. At least one clinically actionable genomic alteration was detected in 332 (95%) patients. According to result reports from testing platforms, 23.2% (*n* = 81) had an FDA approved targeted therapy available for their tumor, 61.3% (*n* = 214) had an off-label FDA approved targeted therapy available, and 77.9% (*n* = 272) were potentially eligible for a clinical trial. A total of 408 treatment recommendations were annotated as FDA-approved or off-label therapies and they were reviewed within the OncoKB database. Forty-seven percent of these did not have any level of evidence assigned. Of the 408 actionable alterations, 7.8% were assigned level 1, 9.8% were assigned level 2, 8.6% were assigned level 3, 17.6% as level 4, and 8.6% as level R1. See Table 2 for a list of all actionable alterations and their corresponding OncoKB levels. 

**Figure 1:**
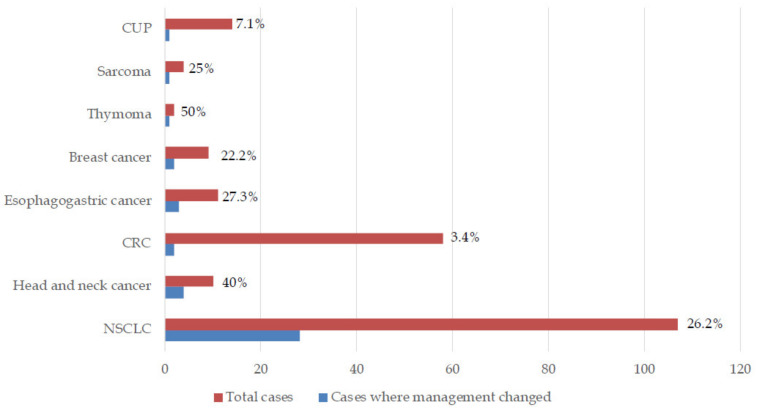
Patients in whom comprehensive genomic profiling changed/potentially changed management (NSCLC, Non-small cell lung cancer, CRC, Colorectal cancer, CUP, Carcinoma of unknown primary).

The patients had a median follow-up of 1.3 years from the date of the reported CGP results. Despite the high number of listed actionable alterations, management was actually changed based on CGP in only 10% (*n* = 35) of patients. In another seven patients, treatment change was planned, but the patient either declined treatment or died prior to its initiation. Of these 42 patients, the most common diagnosis was NSCLC (*n* = 28). CGP-driven management change was observed in 50% (*n* = 1/2) of thymoma, 40% of head and neck cancer (*n* = 4/10), 26.2% (*n* = 28/107) of NSCLC, 27.3% (*n* = 3/11) of esophagogastric, 25% (*n* = 1/4) sarcoma, 22.2% (*n* = 2/9) of breast cancer, 7.1% (*n* = 1/14) carcinoma of unknown primary, and 3.4% (*n* = 2/58) of CRC patients. Figure 1 shows patients in whom management changed categorized by diagnoses. We compared the effect of CGP on the two most common patient diagnoses i.e., lung and colorectal cancer using the Chi-squared test. CGP-led management change was significantly higher in lung cancer versus colorectal patients (*p* = 0.0002). Table 3 describes patient information and clinical outcomes of patients where CGP changed management. Nineteen patients (54% of those treated and 5% of total) had documented clinical benefit with targeted therapy based on CGP. Only one patient received immunotherapy based on CGP testing identifying Microsatellite Instability–High (MSI-H) status. It should be mentioned that, given routine Mismatch Repair (MMR) testing at our institution for all colorectal and endometrial cancers, this low frequency is expected.

Of the 81 patients with FDA approved targeted therapies identified by testing platforms, 55 (67.9%) did not result in change in management. Twelve of these had previously known mutations in NSCLC/CRC/gastric/breast cancer as part of established first tier reflex testing. In 37 patients, the recommended targeted therapy was not standard of care, including high tumor mutational burden directed immunotherapy, four patients had early disease where targeted therapy was not indicated, in one patient therapy was reserved for future disease progression, and one patient died. Although 54 patients (15.5%) participated in clinical trials, only five were enrolled in clinical trials based on results from CGP (including two patients on NCI-MATCH).

## 4. Discussion

In our study, CGP identified at least one potentially clinically actionable genomic alteration in 95% of patients, which is similar to several other reports [1,10,21]. However, management changed based on these results in only 10% of patients. An additional 2% who could have benefited were unable to, as they either died prior to its initiation or declined therapy. Although our numbers were too small to draw any robust conclusion on response rates, 19 patients (54% of those treated and 5% of total) had documented clinical benefit with targeted therapy that was based on CGP, which is similar to that recently predicted by Marquart et al. [18]. Most of the patients who benefited were those who had NSCLC, which was also the most common patient diagnosis. When comparing lung cancer to the next most common diagnosis, colorectal cancer; GCP-led management change was significantly higher in the former (*p* = 0.0002). 28 of 107 (26.2%) NSCLC patients had change/potential change in management based on CGP. As a result of first tier Epidermal Growth Factor Receptor (EGFR)/Anaplastic Lymphoma Kinase (ALK) testing, the majority of EGFR/ALK mutated patients were identified outside of CGP testing. If such first-tier testing results were to be included, the impact of molecular testing would be much higher. In addition, recent studies, for example, with K-Ras G12C inhibitors offer the hope of further expansion of actionable targets [22]. A recent study utilized a decision analytic tool to compare upfront CGP vs. sequential testing for genomic alterations in metastatic NSCLC patients [23]. The study found that upfront CGP led to the same (as panel) or shorter (vs. sequential testing) turnaround time and lowest payer cost in these patients and it is likely to become the preferred approach in most institutions. 

In other cancer types in our study, the impact was much less, soberingly with only 14 of 242 patients (5.8%) with change/potential change in management based on CGP results. Again, the impact would be higher if first-tier testing such as *HER2* in breast/upper GI and extended-*RAS* in colorectal cancer are included. The impact of CGP will likely also increase with the tissue/site agnostic approval of pembrolizumab in solid tumors with microsatellite instability and Tropomyosin Receptor Kinase (TRK) inhibitors for Neurotrophic Tropomyosin-Related Kinase (NTRK)-translocation positive malignancies; however, the frequency of these alterations admittedly is low [7,24]. Tumor mutational burden (TMB) as a predictive biomarker for immune checkpoint inhibitors is another emerging use of CGP and it might add to its impact in routine use in clinical practice, although initial results in the context of advanced NSCLC have been disappointing [25,26]. 

Despite the aforementioned emerging uses of CGP, at present, data to support therapeutic recommendations for many targeted drugs is not robust enough to warrant therapy off a clinical trial, e.g., trastuzumab in (v-erb-b2 avian erythroblastic leukemia viral oncogene homolog 2) ERBB2 amplified lung cancer. Our review of treatment recommendations in the OncoKB database showed that 47% of them did not have strong clinical evidence to support their use. Only 17.6% of recommendations were based on level 1 or 2 evidence. Therefore, the actual actionability of these genomic alterations is currently significantly less than presented. Additionally, an increasing number of commercial as well as institutional multi-gene panels are now being utilized for CGP; however, there is no standardization of therapeutic recommendations based on results [27]. Multidisciplinary molecular tumor boards can certainly help interpret results of CGP, especially in cases where a clinician needs to decide on whether or not to start a patient on an off-label therapy [28,29]. While currently tissue-based CGP utilizing panels, such as ones used in our study, are the most widely utilized, admittedly, further technological advances in circulating tumor DNA (ctDNA), circulating tumor cells (CTC) technology, and the incorporation of whole exome sequencing (WES) and whole genome sequencing (WGS) will provide expansion of information as to tumor heterogeneity, clonal evolution, dynamic assessment of treatment response, and minimal residual disease status that will better inform clinical decision making [30,31,32,33,34,35,36].

Our study has certain limitations, particularly because of its retrospective nature. The timing and choice of CGP panel was not standardized and it was at the discretion of the treating oncologist; however, this indeed best captures a “real-world” scenario in the community as compared to other publishes studies. Another drawback is the short follow-up and the proportion of patients with change in management based on CGP would likely increase with time, due to disease progression or more targeted agents becoming standard of care. We did not include patients who benefited from targeted therapy based on first-tier testing. However, this study specifically assessed the impact of CGP in settings such as ours, where there might be a two-step process for genomic profiling, likely representing the majority of practice patterns both in and especially outside of the United States. Another issue is optimal timing for CGP. Whereas some patients with actionable genetic alterations had early stage disease and, hence, not initiated on targeted therapy, some deteriorated clinically prior to the initiation of treatment. This makes a case for obtaining CGP as soon as possible with metastatic disease or locally advanced disease with a high risk of recurrence. Serial liquid biopsies are now being utilized for the real-time assessment of tumor mutations and more studies are needed to inform the decision of ideal timing for CGP. The lack of available molecularly driven protocols was not a factor, with more than 200 open clinical trials, including the NCI-MATCH trial actively recruiting patients at our institution. Moreover, our enrolment rate of 15% was higher than the national average indicating the robustness of our clinical trials program [37]. Our study focused on patients with whom a change in management was possible as a result of CGP. However, we are unable to comment whether such a treatment approach indeed has merit. We realize that the ultimate litmus test of CGP based treatment is to prove that treating a patient based on a specific mutation is actually superior than offering a non-molecularly targeted agent, either on or off a clinical trial. One randomized trial attempting to answer this question is the Therapy Based on Tumor Molecular Profiling Versus Conventional Therapy in Patients With Refractory Cancer (SHIVA) study, which did not show benefit of the molecularly directed therapeutic approach [38]. Recent studies add to this database and offer more promise as to the benefit of CGP in this context [39]. 

## 5. Conclusions

Overall, our study provides a real-world experience of the impact of CGP in a community-based academic NCI-designated cancer center serving a highly diverse patient population, where molecular testing is based on a two-tier testing algorithm. While recognizing that a 10% overall rate of management change that is based on CGP is very modest, its use in certain subsets, such as advanced NSCLC, where the impact currently is most significant appears to be justifiable and it has been found to be cost effective [40,41,42]. In addition, we have identified multiple reasons for the relatively smaller clinical impact of CGP in other tumor types, despite a much larger proportion of patients with actionable genomic alterations reported. The impact however is anticipated to be increasing in light of new advances, e.g., recent studies do suggest expanding impact in breast (PI3kinase inhibitors) and pancreatic malignancies (poly ADP ribose polymerase (PARP) inhibition) [43,44,45,46,47]. Although issues, such as optimal timing, access to clinical trials, and consolidating genomic testing need to be addressed at an institutional level, reports from testing platforms need to be carefully interpreted and ideally discussed in molecular tumor boards to provide the best treatment option possible. More prospective trials are needed that would better inform our choices for personalized treatment by providing assessments of overall survival and quality of life with choosing targeted therapies that are based on CGP when compared to conventional therapies [11]. 

## Figures and Tables

**Table 1:** Primary diagnosis for patients (Appendix, Online only).

Type of Tumor	Number	Type of Tumor	Number
NSCLC	107	Gastric cancer	5
Colon cancer	47	Sarcoma	4
Ovarian cancer	15	Cervical cancer	4
Carcinoma of unknown primary	14	Myelodysplastic syndrome	4
Pancreatic cancer	11	Hepatocellular carcinoma	3
Uterine cancer	11	Thyroid cancer	3
Head and Neck cancer	10	Lymphoma	3
Renal cancer	9	Pancreatobiliary cancer	3
Breast cancer	9	Parotid cancer	3
Brain tumors	8	Multiple myeloma	2
Prostate cancer	7	Small cell lung cancer	2
Gallbladder cancer	7	Thymoma	2
Rectal cancer	11	B-ALL	2
Bladder cancer	6	Melanoma	2
Esophageal cancer	6	Germ cell tumors	2
Cholangiocarcinoma	5	Others	25

**Table 2:** Classification of actionable molecular alterations according to OncoKB levels of evidence (of note, some alterations had more than one level of evidence assigned depending on the alteration and specific therapy involved).

OncoKB Level of Evidence	Number	Percentage
Level 1	32	7.8
Level 2A	9	2.2
Level 2B	31	7.6
Level 3A	4	0.9
Level 3B	31	7.6
Level 4	72	17.6
Level R1	35	8.6
No level assigned, oncogenic	71	17.4
No level assigned, likely oncogenic	57	14.0
No level assigned, oncogenic function unknown	39	9.6
No level assigned, no information available	8	2.0
No level assigned, Tumor mutational burden	8	4.4
No level assigned, Microsatellite Instability	18	0.2

**Table 3:** Observed/proposed change in management based on comprehensive genomic profiling (*Documented response or on treatment for at least 3 months).

	Diagnosis	Molecular Alterations	Management Change	Observed Benefit
1.	Lung adenocarcinoma	EML4-ALK fusion (Variant 1)	Crizotinib	Lost to follow-up
2.	Lung adenocarcinoma	EGFR amplification, G719A	Erlotinib continued	Yes
3.	Lung adenocarcinoma	EGFR G719A, Q701L, amplification	Erlotinib continued	Yes
4.	Lung adenocarcinoma	EGFR E746_A750del MET amplification	Crizotinib-Erlotinib	Lost to follow-up
5.	Poorly differentiated NSCLC, sarcomatoid morphology	NTRK1 TPM3-NTRK1 fusion	Died prior to giving Crizotinib	N/A
6.	Lung adenocarcinoma	EGFR amplification, exon 19 deletion	Afatinib	Yes
7.	Lung adenocarcinoma	EGFR exon 19 deletion, T790M	Osimertinib	Yes
8.	Lung adenocarcinoma	ALK EML4-ALK fusion (Variant 1)	Alectinib	Yes
9.	Lung adenocarcinoma	BRCA2 S1099* MET amplification	Declined participation in MATCH study	N/A
10.	Lung adenocarcinoma	EGFR L858R	Died prior to starting EGFR-TKI	N/A
11.	Lung adenocarcinoma	EGFR exon 19 deletion, T790M, L792F, C797S	Osimertinib continued beyond progression	N/A
12.	Medullary thyroid cancer	RET V804M	Cabozantinib Lenvatinib Phase 1 study of MGCD516	Yes
13.	Poorly differentiated NSCLC	MET amplification	Died prior to planned phase 1 trial of MGCD516	N/A
14.	Poorly differentiated NSCLC	MET amplification	Died prior to planned phase 1 trial of MGCD516	N/A
15.	Lung adenocarcinoma	EGFR exon 19 deletion (L747_S752del) T790M	Osimertinib	Yes
16.	Lung adenocarcinoma Urothelial bladder cancer	Numerous	Clarified primary tumor to be urothelial in origin	Yes
17.	Gastric adenocarcinoma	MSI-High	Pembrolizumab	Yes
18.	Lung adenocarcinoma	EGFR exon 19 deletion (E746_A750del), T790M	Osimertinib	Yes
19.	Lung adenocarcinoma	EGFR amplification, L858R, R776C, T790M MET amplification	Osimertinib + crizotinib	Yes
20.	Lacrimal duct carcinoma	ERBB2 amplification	Trastuzumab	No
21.	Nasopharyngeal adenoid cystic carcinoma	PIK3CA H1047R	Taselisib on MATCH study	Yes
22.	Lung adenocarcinoma	EGFR exon 19 deletion	Erlotinib after clearance of T790M	No
23.	Thymoma	CDKN2A/B loss KDM6A W1194*	Phase I/IIa trial of ALRN-6924 in patients with wild typeTP53	Yes
24.	Invasive ductal breast cancer	CCND1 amplification	Abemaciclib	No
25.	Lung adenocarcinoma	RET KIF5B-RET fusion, RET-KIF5B fusion	Phase 1/1b MGCD516	Not documented, patient withdrew from study
26.	Lung adenocarcinoma	BRAF V600E	Vemurafenib Dabrafenib +Trametinib	Yes
27.	Lung adenocarcinoma	EGFR exon 19 deletion	Erlotinib after clearance of T790M	No
28.	Lung adenocarcinoma	EGFR amplification, exon 19 deletion (E746_A750del), T790M	Osimertinib	Yes
29.	Rectal adenocarcinoma	ERBB2 amplification, V777L	Ado-trastuzumab emtansine on MATCH study	Yes
30.	Esophageal adenocarcinoma	ERBB2 amplification	Trastuzumab	Patient lost to follow-up/did not complete treatment
31.	Carcinoma of unknown primary, likely upper GI/pancreaticobiliary origin	MET amplification	Planned for crizotinib but not approved by insurance	N/A
32.	Lung adenocarcinoma	EGFR amplification, L858R, T790M	Osimertinib	Yes
33.	Invasive ductal breast cancer	CCND1 amplification	Palbociclib	No
34.	Lung adenocarcinoma	EGFR exon 19 deletion	Afatinib	Yes
35.	Salivary ductal carcinoma	VEGFA amplification	Sorafenib	No
36.	Colon adenocarcinoma	Numerous	Pembrolizumab based on numerous mutations detected and concern for MSI status	No
37.	Esophageal squamous cell carcinoma	EGFR amplification	Panitumumab	No
38.	Lung adenocarcinoma	ALK EML4-ALK fusion (Variant 3a/b)	Alectinib	Yes
39.	Lung adenocarcinoma	EGFR L964L	Erlotinib	Yes
40.	Follicular dendritic cell sarcoma	AKT2 amplification	Everolimus	Yes
41.	Lung adenocarcinoma	MET H1094R	Died prior to starting crizotinib	N/A
42.	Lung adenocarcinoma	MET exon 14 splice site (D1010N)	Crizotinib	No

(EGFR, Epidermal growth factor receptor; MATCH, Molecular Analysis for Therapy Choice; TKI, Tyrosine Kinase Inhibitor; MSI, Microsatellite Instability). VEGF-A, Vascular endothelial growth factor A; NSCLC, Non-Small-Cell Lung Cancer; ALK, anaplastic lymphoma kinase; EML4, echinoderm microtubule-associated protein-like 4.
